# ANTEROMEDIAL OR CENTRAL ANATOMIC ACL RECONSTRUCTION? A CADAVERIC HIP-TO-TOE STUDY

**DOI:** 10.1590/1413-785220233104e268195

**Published:** 2023-07-31

**Authors:** TIAGO LAZZARETTI FERNANDES, MICHEL OLIVEIRA SOUZA, CYRO ALBUQUERQUE, PAULO HENRIQUE ARAUJO, ANDRE PEDRINELLI, ARNALDO JOSÉ HERNANDEZ

**Affiliations:** 1 Universidade de Sao Paulo, Faculdade de Medicina, Hospital das Clinicas, Instituto de Ortopedia e Traumatologia IOT HCFMUSP, Grupo de Medicina Esportiva, Sao Paulo, SP, Brazil.; 2 FIFA Medical Center of Excellence, Sao Paulo, SP, Brazil.; 3 Hospital Sirio-Libanes, Sao Paulo, SP, Brazil.; 4 Centro Universitario da Fundaçao Educacional Inaciana “Padre Saboia de Medeiros”, Departamento de Engenharia Mecanica, Sao Bernardo do Campo, SP, Brazil.

**Keywords:** Anterior Cruciate Ligament, Anterior Cruciate Ligament Reconstruction, Cadaver, Ligamento Cruzado Anterior, Reconstrução do Ligamento Cruzado Anterior, Cadáver

## Abstract

**Objective:**

To compare anatomic anterior cruciate ligament (ACL) reconstruction between two tunnel positions in knees with isolated ligament tears.

**Methods:**

Anatomic ACL reconstruction was performed, from hip-to-toe, on 15 fresh cadaveric specimens. No associated lesions were created to enhance knee instability. The protocol was conducted in three states: (1) complete isolated ACL deficiency; (2) anatomic femoral and tibial anteromedial ACL reconstruction (AM REC); and (3) anatomic femoral and tibial central ACL reconstruction (Central REC). The reconstruction protocols were randomly assigned. The continuous mechanized pivot-shift test was recorded dynamically with a tracking system.

**Results:**

The Central REC group showed a smaller degree of internal rotation (0.6° ± 0.3° vs. 1.8° ± 0.3°, respectively, P < 0.05) and no difference in anterior translation (4.7 mm ± 0.4 mm vs. 4.5 mm ± 0.4 mm, respectively, P > 0.05) in the pivot-shift test, compared with the AM REC group.

**Conclusion:**

The central anatomic ACL reconstruction resulted in greater restriction of internal rotation than the anteromedial anatomic ACL reconstruction. ***Experimental Study on Cadaver.***

## RESUMO

**Objetivo:**

Comparar a reconstrução anatômica do ligamento cruzado anterior (LCA) entre duas posições de túnel em joelhos com lesões isoladas do ligamento.

**Métodos:**

A reconstrução anatômica do LCA foi realizada, do quadril aos pés, em 15 peças anatômicas de cadáveres frescos. Não foram criadas lesões associadas para intensificar a instabilidade do joelho. O protocolo foi realizado em três estados: (1) deficiência isolada completa do LCA; (2) reconstrução anatômica femoral e anteromedial tibial do LCA (AM REC); e (3) reconstrução anatômica femoral e central tibial do LCA (Central REC). Os protocolos de reconstrução foram atribuídos aleatoriamente. O teste de pivot-shift mecanizado contínuo foi registrado dinamicamente com um sistema de rastreamento.

**Resultados:**

O grupo Central REC apresentou menor grau de rotação interna (0,6° ± 0,3° vs. 1,8° ± 0,3°, respectivamente, p < 0,05) e nenhuma diferença na translação anterior (4,7 mm ± 0,4 mm vs. 4,5 mm ± 0,4 mm, respectivamente, p > 0,05) no teste de pivot-shift, comparado ao grupo AM REC.

**Conclusão:**

A reconstrução anatômica central tibial do LCA resultou em maior restrição da rotação interna do que a reconstrução anteromedial tibial do LCA. **Estudo em Cadáver Experimental.**

## INTRODUCTION

The pivot-shift test is the focus of basic and clinical research in the evaluation of knee ligament surgeries.^1^ ^),(^ ^2^ Knee kinematics during the pivot-shift test may represent the most clinically relevant biomechanical outcome when comparing surgical techniques for reconstruction of anterior cruciate ligament (ACL).^3^

The concept of ACL reconstruction is constantly changing and there is no consensus on the best anatomical position of the tunnel.^4^

This study mainly aimed to compare knee stability in ACL reconstruction between two different anatomical positions of the tibial tunnel (anteromedial and central) in anatomical pieces of cadavers from hip to foot after an isolated ACL rupture.

It was hypothesized that the anatomical reconstruction of the ACL performed in the middle of the original impressions of the femoral and tibial ACL should be more effective in controlling the kinematics of the internal rotation of the knee than that performed in the anatomical impression of the anteromedial bundle.

## METHODS

### Protocol

A total of 15 anatomical pieces of lower extremities of fresh male cadavers, from hip to foot, aged 65.3 ± 9.8 years (mean ± standard deviation) were used. The set of tests described was performed on each of the 15 knees in three states, including (1) without the ACL (ACL-absent); (2) ACL reconstruction with femoral and central anatomical tunnel in the tibia (Central REC), and (3) ACL reconstruction with femoral and anteromedial anatomical tunnel in the tibia (AM REC). The AM REC and Central REC were performed in random order to reduce the risk of lateral condyle wall rupture bias.

The fresh samples were kept in a refrigerator at 4 °C and the procedures were performed at 16 °C (room temperature). Each specimen was placed in dorsal decubitus and the pelvis was fixed on the operating table to allow external load and free and unrestricted range of motion of the hip and knee.

Each measurement was performed at least three times to ensure high repeatability of the pivot-shift pattern, and the first measurement was used for analysis and comparisons.

Our institutional review board approved this study, and permission was obtained from the Research Ethics Committee of the University of São Paulo (CEP No. 436/11).

No soft tissue was cut or removed from the area around the knee or adjacent joints, which would amplify knee instability.

In total, 30 ACL reconstruction procedures were performed under anatomical conditions and were randomized to AM or central tunnel positioning surgery using a randomization plan generator.

Individuals without any significant deformity and surgical intervention were selected and examined manually. A standard anteromedial arthrotomy was performed to verify the ligamentous integrity of the joint and the presence of meniscal and gross lesions of the articular cartilage, bone abnormalities, and osteoarthritis. Knees with any of these signs were excluded from the study.

### Surgical technique

The same surgeon performed all ACL reconstructions. A five-centimeter medial parapatellar arthrotomy was created in each knee.

The remnants of the ACL footprint on the femoral and tibial sides were used to indicate the tunnel positions. The AM REC was performed by passing the graft through the anatomical femoral tunnels and at the site of the anteromedial band of the ACL in the tibia, and the Central REC was performed by passing the graft through the anatomical femoral tunnels and in the middle of the ACL bands in the tibia (central).

The anterior tibial tendon was removed from the ankle of the opposite limb. The loop of the tendon created a double-stranded graft, and an 8 mm diameter graft was standardized for all surgical procedures.

**Figure 1:**
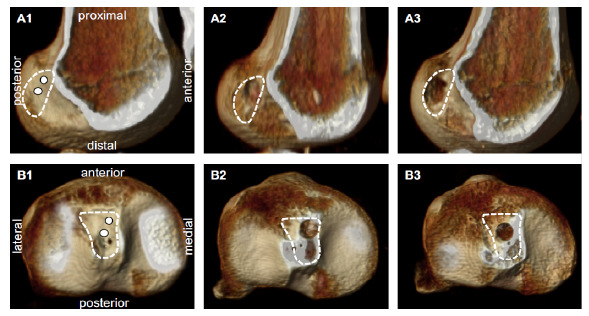
Rendered 3D computed tomography of the anatomical reconstruction of the anterior cruciate ligament. A1 and B1: representative position of the anteromedial tunnel (upper white dot) and central tunnel (lower white dot) in the femur and tibia, respectively; A2 and A3: anteromedial and central tunnels in the femur, respectively; B2 and B3: anteromedial and central tunnels in the tibia, respectively.

The femoral and tibial tunnels were drilled into the anatomical footprint using an outside-in technique, depending on randomization. Femoral and tibial fixations were performed with a radiolucent and bioabsorbable screw (9 mm × 28 mm, Biosteon^®^ HA/PLLA, Stryker, USA), and the impact of the intercondylar notch in full extension was verified before tibial fixation. Notchplasty was not performed (Figure 1).

Before fixation, each limb was preconditioned with 10 flexion-extension cycles from 0° to 130°. The graft was manually tensioned and fixed to the tibia in the knee extension position with an interference screw while a manual posterior tibial load was applied. After fixation, 10 flexion-extension cycles were performed to accommodate the graft.

After biomechanical examinations and computed tomography (CT) for the first ACL reconstruction, the screws and tendon graft were removed.

Donor bone plugs that were 1 mm larger than the tunnel size were harvested from the extra-articular side of the medial condyle using a 10 mm osteochondral donor plug collector (Arthrex, Naples, FL) and pressure adjustment to completely fill the previously used tunnels.^3^

The second ACL tunnel was then drilled randomly as described earlier. The walls of the new tunnels were probed before and after the tests to ensure their integrity. No cortical fractures or ruptures of the lateral condyle were observed.

The same undamaged tendon grafts were used for the second reconstruction, and each reconstruction used the same fixation methods.

The graft was passed, tensioned, and fixed following the same procedure as the first reconstruction. The test protocol described previously was performed.

### Mechanized Pivot-Shift

An instrumented pivot-shift test was performed using a continuous passive motion (CPM) machine (Carci, Ortomed 4060, ANVISA: 10314290029) that has been fixed to the operating table. A custom-made foot support was attached to allow the application of an internal rotation moment in the knee and axial load.^5^

This machine was developed at the Biomechanics Laboratory of the Institute of Orthopedics and Traumatology (IOT HCFMSUP) and was compatible with a device described by Musahl et al.^6^

The pivot-shift examination technique followed the description of Galway and MacIntosh.^7^ The leg was flexed from a fully extended position with an axial load while a valgus and internal rotation moment was applied to the leg.

**Figure 2:**
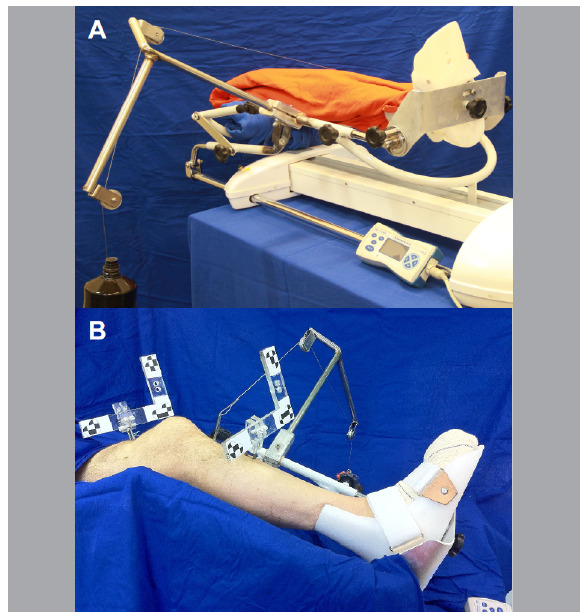
A: Mechanized pivot-shift with cable and pulley system for valgus and internal rotation moments. Note the foot support with internal rotation of 15° (CPM, Carci, Ortomed 4060); B: Reflective markers on the tibia and femur for the optical tracking system.

A cable and pulley system was used to perform a valgus and internal torque moment with a 45° inclination in relation to the operating table. This was consistent with the procedure described by Musahl et al.,^6^ in which the tibia was subluxated anteriorly in relation to the femur (Figure 2).

A 20 Nm torque was applied to a 15 cm Steinmann pin that was fixed vertically to the tibial tuberosity.

The thigh supports were removed, and the femur was completely loosened to allow free movement of the hip and knee. The tibia was fixed in its position on the foot support.^6^

The CPM machine moved the knee from full extension to 55° of flexion dynamically and in a multidirectional motion,^8^ a navigation system simultaneously recorded the kinematics of the knee frame by frame (15 Hz).

### Tracking system

A computer-aided navigation system was used to evaluate the knee kinematics and allowed the decomposition of the pivot-shift and Lachman tests.

Two Steinmann pins (2.5 mm) were placed in the anterior cortical of the distal femur and proximal tibia, where the rigid bodies were fixed approximately 10 cm away from the joint line. Each rigid body had a distinct configuration of reflective markers that could be tracked by an optical locator (Figure 2).

A bifocal tracking camera (MicronTracker 2; model H40; Toronto, Canada; 15 Hz; manufacturer’s accuracy of 0.2 mm) was used to track the optical markers on the rigid bodies. A routine (Basic SQL) was created to recognize and save 3D data (X, Y, Z) in real time (15 Hz, 0.2 mm accuracy).^9^

Data acquisition involved calibration, rigid body recording, and a movement sequence to create accurate dynamic models of knee movement with 6° of freedom (calculated relative standard error = 0.82% from 350 to 800 mm).

Radiopaque markers in the same position were scanned using CT and were used to align and merge the optical tracking and three-dimensional CT systems. The anterior translation of the tibia and the internal rotation were expressed in millimeters and degrees, respectively.^9^

### Coordinate system

The 3D models of bone scans (axial thickness of 1 mm; CT Emotion 2010; 16 channels; Siemens; PISA Project) were digitized and processed according to the descriptions provided by Chen et al.^10^ and Van de Velde et al.^11^

The condyle geometric axis was used to create the femoral coordinate system. The tibial coordinate system was defined by the mechanical axis and centroids of the ellipses embedded in the medial and lateral tibial plateaus (Rhinoceros^®^, McNeel, Seattle, WA).^10^ ^),(^ ^11^

Internal and external rotation were measured according to the classic study by Grood and Suntay,^12^ and anteroposterior motion was defined as the displacement of the center of the femoral coordinate system in relation to the tibial coordinate system in the anterior direction.^10^ ^),(^ ^11^ ^),(^ ^13^

### Evaluation of the position of the ACL tunnel

Postoperative tunnel positions were evaluated using a rendered 3D CT protocol.

For the femur, tunnel positioning was measured according to the method of Bernard et al.,^14^ who described the position of the center of the tunnel as a percentage of the distance along the Blumensaat line (from proximal and posterior to distal and anterior) and as a percentage of the distance along a line perpendicular to the Blumensaat line (from proximal and anterior to distal and posterior).

The positioning of the tunnel in the tibia was measured according to the method of Lorenz et al.^15^ and consisted of a percentage of the height of the tibial plateau (vertical axis) and length (horizontal axis).

### Statistical analysis

The sample size was calculated based on the first five experiments for the primary outcome, internal tibial rotation (mechanized pivot-shift between the AM REC and the Central REC). The minimum difference in the mean was 1.92° and the standard deviation was 1.42°. The sample size was 15 (groups = 4, alpha = 0.05, power = 0.80, sample size for ANOVA, SigmaPlot 12.5).

Kinematic data based on the results of pivot-shift loading tests were analyzed using 2-way RM-ANOVA and a post hoc multiple comparison test. Significance was set at P < 0.05 (SigmaPlot 12.5).

The statistical power of the study was calculated based on the final data for four groups of 15 subjects with alpha equal to 0.05. The minimum difference in the mean was 1.5° and the standard deviation was 1.1°. The statistical power of the study was equal to 85.5% (Power for ANOVA, SigmaPlot 12.5).

## RESULTS

**Figure 3:**
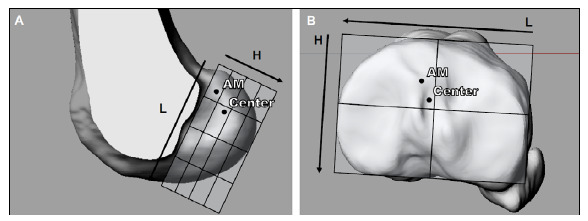
A: the method of Bernard et al.^14^ to measure the position of the femoral tunnel of the anterior cruciate ligament for anteromedial (upper point) and central anatomical (lower point) reconstructions of the anterior cruciate ligament; B: the method of Lorenz et al.^15^ to measure the position of the tibial tunnel of the anterior cruciate ligament for anteromedial (upper point) and central anatomical (lower point) reconstructions.

Figure 3A shows the mean and standard deviation of femoral tunnel positioning for anatomical AM (length: 20.8% ± 5.7%; height: 27.0% ± 11.6%) and central (length: 39.5% ± 5.1%; height: 52.4% ± 9.6%) ACL reconstructions according to the quadrant method of Bernard et al.^14^ A baseline analysis showed that the AM and central tunnel positions differed significantly (P < 0.001).

L: length of the tibial plateau, H: height of the tibial plateau (Rhinoceros^®^, McNeel, Seattle, WA).

Figure 3B the mean and standard deviation of tibial tunnel positioning for anatomical AM (length: 56.4% ± 4.1%; height: 30.6% ± 4.3%) and central (length: 51.4% ± 2.4%; height: 43.2% ± 5.7%) ACL reconstructions according to the method of Lorenz et al.^15^ A baseline analysis showed that the AM and central tunnel positions differed significantly (P < 0.001).

**Figure 4:**
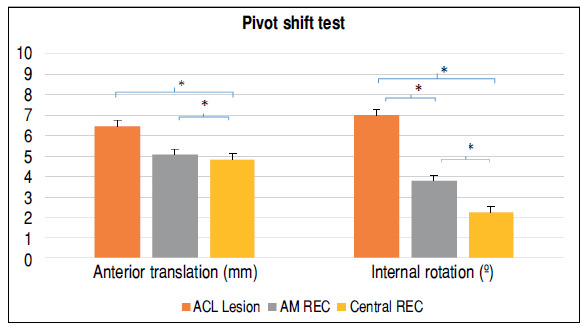
The comparisons of the pivot-shift test of the knee kinematics between the groups absence of anterior cruciate ligament (red), femoral and anteromedial tibial anatomical reconstruction of the anterior cruciate ligament (gray), and femoral and central tibial anatomical reconstruction of the anterior cruciate ligament (yellow).

Figure 4 shows the results of the instrumented pivot-shift test.

AM REC: anteromedial anatomical reconstruction of the ACL; Central REC: central anatomical reconstruction of the ACL.

Two-way RM-ANOVA, * P < 0.05.

## DISCUSSION

The most important finding of this study was that the Central REC produced a lower degree of internal rotation than the anatomical AM REC based on the mechanized pivot-shift test, which partially confirmed our initial hypothesis.

Diermann et al.^1^ stated that an ACL deficiency leads to increased internal rotation of the tibia in a simulated pivot-shift test and that the anatomical reconstruction of the single-bundle ACL significantly reduces internal tibial rotation in a simulated pivot-shift test when compared with an absent ACL.

Our study found that a simulated pivot-shift test resulted in more significant anterior translation of the tibia, but not internal rotation in the group with ACL absence.

These results are in line with other studies that have used a robotic test system.^1^ ^),(^ ^7^

All secondary stabilizers were preserved. We used an anatomical piece with lower limb from hip to foot. A possible explanation for the difference obtained by Diermann et al.^1^ regarding internal rotation in the group with ACL injury is that soft tissue resection and the use of small pieces of bone may have increased knee instability.

The resulting anterior tibial translation and internal rotation were evaluated for the first time by using a simulated pivot-shift test in a complete cadaver model from hip to foot without associated injuries to amplify knee instability.

Regarding the use of intentionally associated injuries to increase knee instability, Cross et al.^16^ stated that meniscus resection undoubtedly influenced knee kinematics after ACL reconstruction when compared with a reconstructed knee with intact meniscus.

Our study used a mechanized device, and the magnitude of the pivot was large enough to detect statistically significant differences between the groups without meniscal resecting.

Differences in the magnitude of mean values between statistically different groups may be a weak point in this study. Although the differences were small between the groups, this study was well designed and properly conducted, increasing internal validity.^3^ ^),(^ ^6^ ^),(^ ^16^ ^),(^ ^17^

The effect size was in agreement with other published biomechanical studies and the power of the study was adequate (85%) to calculate small differences for the primary outcome, which reduced the potential bias of a type II error.^3^ ^),(^ ^6^ ^),(^ ^16^ ^),(^ ^17^

The device used in this study is simpler than robotic systems and can achieve consistent and observer-independent results.

The results of our study show that the pivot-shift device accurately collected data and replicated the physiological movements of the knee pivot-shift, as discussed by Driscoll et al.^17^

Pearle et al.^18^ validated this model as a reliable tool to quantify knee stability by comparing it with a robotic force-moment test system and sensor. 

According to some studies, the subluxation/reduction event occurs at approximately 20 to 35° of flexion.^6^ ^),(^ ^19^ ^),(^ ^20^ Bedi et al.^3^ state that the maximum displacement occurs at a flexion angle of 10 to 20°. Our study identified similar values for a reduction of subluxation at 30°.

### Limitations

This experiment suffered the disadvantages of using anatomical parts of older adults cadavers *in vitro*, which were much older than the average age at which ACL injuries occur. In addition, the analysis refers to a zero-time condition, and laxity was not influenced by *in vivo* graft relaxation and remodeling.

## CONCLUSION

The main conclusion is that ACL central anatomical reconstruction results in greater restriction of internal rotation than ACL anteromedial reconstruction.

## References

[1] Diermann N, Schumacher T, Schanz S, Raschke MJ, Petersen W, Zantop T (2009). Rotational instability of the knee: internal tibial rotation under a simulated pivot shift test. Arch Orthop Trauma Surg.

[2] Musahl V, Griffith C, Irrgang JJ, Hoshino Y, Kuroda R, Lopomo N (2016). Validation of quantitative measures of rotatory knee laxity. Am J Sports Med.

[3] Bedi A, Musahl V, O&apos;Loughlin P, Maak T, Citak M, Dixon P, Pearle AD (2010). A comparison of the effect of central anatomical single-bundle anterior cruciate ligament reconstruction and double-bundle anterior cruciate ligament reconstruction on pivot-shift kinematics. Am J Sports Med.

[4] Araujo PH, Kfuri M, Ohashi B, Hoshino Y, Zaffagnini S, Samuelsson K (2014). Individualized ACL reconstruction. Knee Surg Sports Traumatol Arthrosc.

[5] Bedi A, Maak T, Musahl V, O&apos;Loughlin P, Choi D, Citak M, Pearle AD (2011). Effect of tunnel position and graft size in single-bundle anterior cruciate ligament reconstruction: an evaluation of time-zero knee stability. Arthroscopy.

[6] Musahl V, Voos J, O&apos;Loughlin PF, Stueber V, Kendoff D, Pearle AD (2010). Mechanized pivot shift test achieves greater accuracy than manual pivot shift test. Knee Surg Sports Traumatol Arthrosc.

[7] Galway HR, MacIntosh DL (1980). The lateral pivot shift: a symptom and sign of anterior cruciate ligament insufficiency. Clin Orthop Relat Res.

[8] Maeyama A, Hoshino Y, Kato Y, Debandi A, Lertwanich P, Wang JH (2018). Anatomic double bundle ACL reconstruction outperforms any types of single bundle ACL reconstructions in controlling dynamic rotational laxity. Knee Surg Sports Traumatol Arthrosc.

[9] Fernandes TL, Ribeiro DB, Rocha DC, Albuquerque C, Pereira CAM, Pedrinelli A, Hernandez AJ (2014). Description of an evaluation system for knee kinematics in ligament lesions, by means of optical tracking and 3D tomography. Rev Bras Ortop.

[10] Chen CH, Li JS, Hosseini A, Gadikota HR, Gill TJ, Li G (2012). Anteroposterior stability of the knee during the stance phase of gait after anterior cruciate ligament deficiency. Gait Posture.

[11] Van de Velde SK, Hosseini A, Kozánek M, Gill TJ, Rubash HE, Li G (2010). Application guidelines for dynamic knee joint analysis with a dual fluoroscopic imaging system. Acta Orthop Belg.

[12] Grood ES, Suntay WJ (1983). A joint coordinate system for the clinical description of three-dimensional motions: application to the knee. J Biomech Eng.

[13] Defrate LE, Papannagari R, Gill TJ, Moses JM, Pathare NP, Li G (2006). The 6 degrees of freedom kinematics of the knee after anterior cruciate ligament deficiency: an in vivo imaging analysis. Am J Sports Med.

[14] Bernard M, Hertel P, Hornung H, Cierpinski T (1997). Femoral insertion of the ACL. Radiographic quadrant method. Am J Knee Surg.

[15] Lorenz S, Elser F, Mitterer M, Obst T, Imhoff AB (2009). Radiologic evaluation of the insertion sites of the 2 functional bundles of the anterior cruciate ligament using 3-dimensional computed tomography. Am J Sports Med.

[16] Cross MB, Musahl V, Bedi A, O&apos;Loughlin P, Hammoud S, Suero E, Pearle AD (2012). Anteromedial versus central single-bundle graft position: which anatomic graft position to choose?. Knee Surg Sports Traumatol Arthrosc.

[17] Driscoll MD, Isabell GP, Conditt MA, Ismaily SK, Jupiter DC, Noble PC, Lowe WR (2012). Comparison of 2 femoral tunnel locations in anatomic single-bundle anterior cruciate ligament reconstruction: a biomechanical study. Arthroscopy.

[18] Pearle AD, Solomon DJ, Wanich T, Moreau-Gaudry A, Granchi CC, Wickiewicz TL, Warren RF (2007). Reliability of navigated knee stability examination: a cadaveric evaluation. Am J Sports Med.

[19] Lane CG, Warren RF, Stanford FC, Kendoff D, Pearle AD (2008). In vivo analysis of the pivot shift phenomenon during computer navigated ACL reconstruction. Knee Surg Sports Traumatol Arthrosc.

[20] Tanaka M, Vyas D, Moloney G, Bedi A, Pearle AD, Musahl V (2012). What does it take to have a high-grade pivot shift?. Knee Surg Sports Traumatol Arthrosc.

